# Mechanisms-based viscoplasticity: Theoretical approach and experimental validation for steel 304L

**DOI:** 10.1038/srep23681

**Published:** 2016-03-30

**Authors:** Aleksander Zubelewicz, Wiera Oliferuk

**Affiliations:** 1 University of New Mexico, Civil Engineering, Albuquerque, NM, USA; 2 Bialystok University of Technology, Bialystok, Poland

## Abstract

We propose a mechanisms-based viscoplasticity approach for metals and alloys. First, we derive a stochastic model for thermally-activated motion of dislocations and, then, introduce power-law flow rules. The overall plastic deformation includes local plastic slip events taken with an appropriate weight assigned to each angle of the plane misorientation from the direction of maximum shear stress. As deformation progresses, the material experiences successive reorganizations of the slip systems. The microstructural evolution causes that a portion of energy expended on plastic deformation is dissipated and the rest is stored in the defect structures. We show that the reorganizations are stable in a homogeneously deformed material. The concept is tested for steel 304L, where we reproduce experimentally obtained stress-strain responses, we construct the Frost-Ashby deformation map and predict the rate of the energy storage. The storage is assessed in terms of synchronized measurements of temperature and displacement distributions on the specimen surface during tensile loading.

Despite significant advances in modeling the inelastic behavior of polycrystalline materials, our understanding of the deformation mechanisms is still incomplete. Temperature, strain rate and strain range alter mechanical properties of the materials, shift the source of the material’s nonlinearity within different levels of the internal structure, thus making the behavior hierarchical in nature^1^. At early stages of plastic deformation, dislocations nucleate, multiply and move freely within grains. As the deformation process continues, grain boundaries and other defects obstruct the dislocation mobility. We know that plastic work is not fully dissipated and a measurable portion of it adds to internal energy of the deformed materials. This energy has been discovered in calorimetric tests performed by Taylor and Quinney^2^. For many years, based on their work, the part of the work converted to internal energy and stored in the metallic material has been considered to be a constant value of about ten percent of the entire plastic work. Further experimental studies have shown that this estimation is wrong^3^,^4^,^5^,^6^,^7^ and, in fact, the ratio of the energy is not constant and depends on the deformation level of the tested material. Consequently, a concept of the energy storage rate is proposed^8^,^9^,^10^,^11^. The stored energy results from the evolution of the microstructure and is an essential measure of its cold work state^12^. Consequently, the microstructural reorganizations increase internal energy of the material. The energy storage is often mitigated by a partial restoration of the grain shape due to the relaxation of dislocation pileups, dynamic recrystallization and/or grain boundary sliding.

Since seventies, constitutive modeling is steadily migrating from purely phenomenological descriptions toward the approaches which are based on micromechanics considerations. The phenomenology means that the models offer reliable responses but only within the domain already verified by experiment. Often, extrapolation of the behavior to the regimes not tested can be troublesome. The introduction of crystal plasticity approaches^13^,^14^,^15^ significantly improves the predictability by correctly specifying the mechanisms of plastic slip. In our approach, we attempt to bridge the benefits of the phenomenology with the advantages of the micromechanics based descriptions. The study consist of four sections. First, we develop a generalized concept of the thermally activated motion of dislocation. Next, we propose a Tresca-type stochastic concept, where the dominant plastic deformation is collected from slip systems placed on the plane defined by the maximum and minimum principal stresses. As a result, we introduce an average Schmid factor. The factor encapsulates the relevant information about the evolving configuration of the active systems. Also, we provide a micromechanisms-based interpretation of the energy storage rate. We point into the conditions, at which the microstructural evolution may become unstable. Lastly, we introduce a visco-plasticity model capable of capturing metal responses within a broad range of strain rates and temperatures.

## Results

### Notation

Dyadic product: **n n** corresponds to *n*_*i*_*n*_*j*_, where in Cartesian coordinate system *i*, *j* = 1, 3

Inner product of second order tensors: 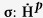 is 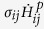


Product of tensors: 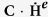 is 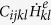


### Thermally activated motion of dislocations

The thermally activated motion of dislocations and subsequent dislocation interactions are reflected in activation free energy 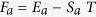, where *E*_*a*_ and *S*_*a*_ are the activation energy and activation entropy^16^. We introduce the non-dimensional function 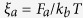, where *k*_*b*_ is Boltzmann constant and *T* is temperature. The processes are stochastic with respect to 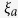 and, therefore, frequency of the dislocation-triggered events follows a statistical distribution and is assumed here to be the Weibull distribution 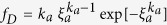. An increase in the entropic contribution *S*_*a*_*T* makes the material softer and strength is reduced. The material is expected to experience the events with a frequency *f*_*D*_(*T*) and with the rate defined by the exponent *k*_*a*_. In a collective sense, the dislocation activities at temperature *T* include all the events available up to this temperature with the appropriate frequencies. These events effectively reduce the material’s resistance to plastic flow. Therefore, the actual resistance *A*_D_ is integrated over the frequencies in the domain taken between *T*_*c*_/*T*_*m*_ and *T*_c_/*T* and, then, we have 
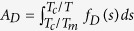
. The variable *s* defines the pathway of temperature *T*_*c*_/*T*. The activation factor *A*_*D*_ is a measure of thermal softening. At temperatures higher from the transition temperature *T*_*c*_ the material becomes increasingly softer and its strain rate sensitivity increases. Often, the transition occurs at temperature close to *T*_*m*_/3, where *T*_*m*_ is melting temperature. In here, the activation energy is expressed in terms of *k*_*b*_*T*_*c*_ such that 
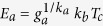
. When entropy is assumed to be weakly dependent on temperature, then at melting point *F*_*a*_(*T*_*m*_) = 0 we have *S*_*a*_ = *E*_*a*_/*T*_*m*_. Consequently, the activation factor becomes


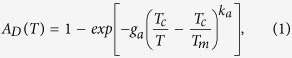


where *g*_*a*_ determines the magnitude of the activation energy. At the solidus temperature, where 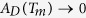, the dislocation activities are replaced by diffusional flow. At very low temperature, the factor *A*_*D*_ is only slightly smaller from one and the material exhibits high resistance to plastic deformation.

### Deformation-induced slip reorganization

In the Tresca-like material, the macroscopically observed plastic strain rate 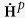 follows the direction of maximum shear stress and is


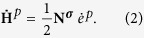


The magnitude of the plastic strain rate 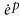 is affected not only by shear stress and temperature but also is sensitive to the evolving slip configuration. Components of the strain rate 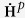 are defined in terms of the dyadic product 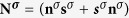. The unite vectors 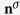 and 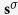 are orthogonal and are determined by the current stress ***σ***. We construct the stress representation 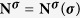, which must retain the properties of the dyadic product 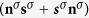, thus 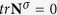, 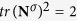 and 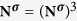. This expression has already been derived^17^ and is


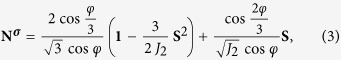


where the angle is 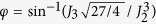. The second and third invariants of stress deviator 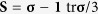 are *J*_2_ and *J*_3_. Note that the expression produces singular responses when the angle *φ* approaches the values ±*π*/2. These points correspond to the corners on the Tresca stress envelop.

Plastic deformation is collected from many slip planes with orientations distributed around the plane of maximum shear stress. Each such *θ*-active plane is defined in terms of orthogonal triad 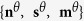, where identity tensor is 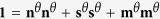 and slip occurs on the planes defined by the unite vectors 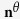 and 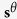. Plastic deformation in each grain is sensitive to the grain orientation and is dependent on the grain size and shape. Incoherencies of the deformation at grain boundaries and in the presence of other defects permanently distort the lattice and are responsible for an increase of internal energy. Consequently, these incoherencies in a plastically deformed material broaden the angular spectrum of the active slip systems. It is worth noting that at large plastic deformation the spectrum tightens again^18^ and the material may experience localized plastic deformation. In here, we narrow the study to the conditions, where the localized shear bands are not present. Further discussion on the conditions, which lead to the formation of shear bands, is presented in the next section. The vectors 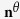 and 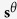 and their rotations occur on the plane defined by the maximum and minimum principal stresses. The 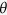-planes undergo rotations such that


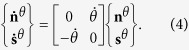


**Figure 1:**
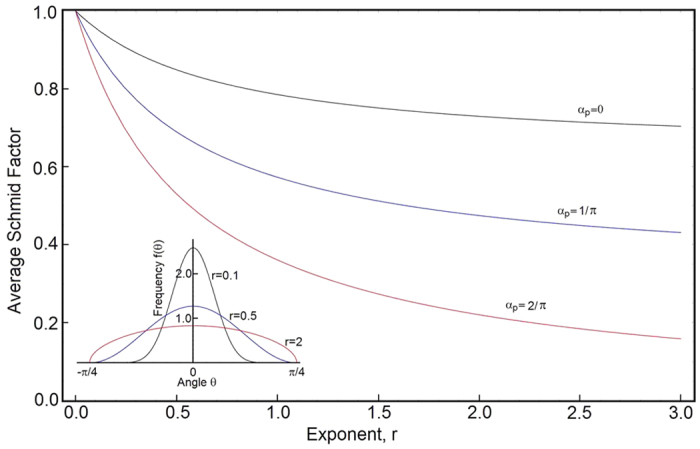
The average Schmid factor *M* specifies flow constraints, which result from the current orientation of slip systems *M*_*s*_ and is sensitive to the reorientations *M*_*r*_. The factor is plotted as a function of the exponent *r* and is shown for three rates of slip reconfigurations 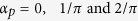. Three shapes of the distribution 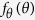 are presented for exponents *r* = 0.1, 0.5 and 2.

The plastic strain rate 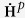 incorporates slip rates from the entire spectrum of the slip orientations, where the slip contributions vary and depend on the current angle 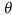. In the representative volume of the materials, we have a large number of such systems and, therefore, we assume that the orientations are continuously distributed about the plane of maximum shear stress. The distribution 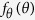 of the slip contributions is taken for angles between ±*π*/4. The form of the distribution is sufficiently general when constructed as follows


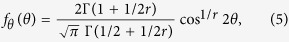


where we assure that 
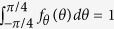
 and 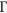 is gamma function. The exponent *r* determines the shape of the distribution. We note that the larger the exponent is the broader the distribution becomes, Fig. 1. At advanced deformation, the slip systems sense each other, interact with grain boundaries and other defects. Consequently, the interactions widen the distribution and create the conditions for plastic hardening. At the microscale, the rate of plastic strain 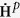 is defined as


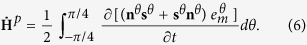


The equivalent microscopic plastic strain 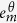 specifies the slip contribution from each orientation 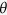. Note that the rate of plastic strain 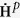 is sensitive to the rates of slip 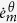 and rotations 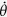; these two mechanisms occur in a plastically deformed material. The reorientations include grain rotations, result from the lattice distortions between closely spaced slip bands, are caused by the activation of secondary slip systems, and there is a possibility that some of the plane become curved^19^. These rotations reflect the material’s ability for overcoming energy barriers, which are caused by the pre-existing and deformation induced defects. In this manner, the rotations are responsible for the plasticity-induced lattice distortions and cause the storage of energy. The rate of the 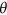-strain is weighted with 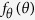, hence 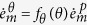. The subscript “m” in 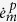 indicates that the equivalent strain rate is taken at the microscale. The reorientations (4) are caused by plastic slip and, therefore, we have 
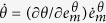
, hence


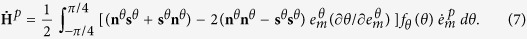


We multiply Eq. 7 by the flow tensor **N**^*σ*^. Also, we note that **N**^*σ*^:**N**^*σ*^ = 2 and, therefore, we compare (2) and (7) and conclude that


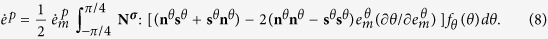


Next, we express 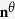 and 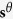 in reference to 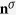 and 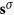, where 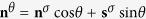 and 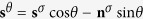 and, then


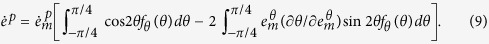


The first term in (9) is the rate of plastic strain collected from the current slip systems. The second term represents the contribution of the slip reorientations. The reorientations broaden the distribution 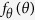 of the available slip systems and, effectively, slow down the flow process. Slip systems that are closely aligned with the direction of maximum shear stress 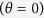 do not rotate^19^,^20^, while systems at the angles 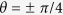 are inactive. As shown^20^, the slip reorganizations are maximized somewhere between 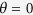 and 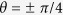. For this reason, we propose expression 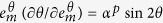. Consequently, the strain-induced reorientations become 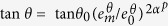, where the initial slip orientation is 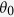 and the reference plastic strain is 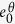. When such a plane is perfectly aligned with the direction of maximum shear stress 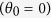, then the plane does not rotate. In the case of the misaligned planes 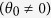, the formula reproduces the experimentally observed trends^20^. The rotations increase misalignment of the planes from the direction of maximum shear stress. We realize that an advanced plastic deformation promotes the conditions for the formation of shear bands. Hence, the active planes become realigned with the band’s orientations. Accordingly, this realignment is responsible for directional plastic flow. We focus the study on the behavior, where the spectrum of the plane distribution experiences broadening and the plastic flow is isotropic. Consequently, the expression (9) becomes


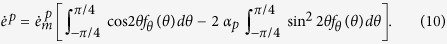


Now, we can write 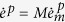, where the average Schmid factor is 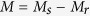. From (10), we have 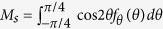 and 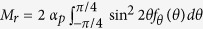. After integrating we obtain


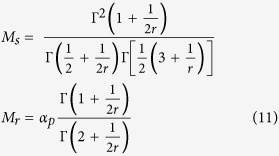


As plastic deformation advances, less favorably oriented systems are activated. The factor *M*_*s*_ quantifies these contributions. The reorientations *M*_*r*_ results from direct interactions of the active systems. The reconfigurations reduce the average Schmid factor and further broaden the spectrum of the plane orientations. When slip is perfectly aligned with the direction of maximum shear stress (*r* → 0), then the average Schmid factor *M* is equal to one and 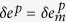, Fig. 1. On the other hand, a uniform distribution of slip contributions 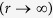 maximizes the flow constraints. The exponent *r* evolves as microscopic plastic strain increases 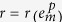. In the simplest scenario, we make the shape of the distribution dependent on temperature and plastic strain and, therefore, we write 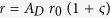 and 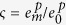 while 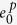 is a material constant. At high temperatures, the activation factor *A*_*D*_ is a small number and, then, the materials exhibits low resistance plastic flow.

### Stability of microstructural evolution

From the requirement of measure invariance (independence from the frame of description) we write 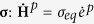, where the equivalent stress 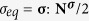 is maximum shear stress. From (10), the rate of plastic work becomes 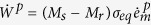. The rate of total plastic work is 
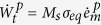
 and the portion 
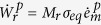
 is retained as internal energy. This mechanism is traditionally known as the stored energy of cold work. The capacity for storing this energy is evolving in the plastically deformed material. In terms of Helmholtz energy, the first order energy perturbation around equilibrium is 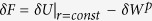, where 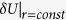 is the change of internal energy calculated in the reference configuration of the slip systems. We narrow down the analysis to conditions, where heat flux can be neglected. Hence, the perturbation becomes


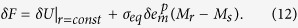


The material exhibits stable microstructural reconfigurations when 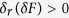. This condition is satisfied as long as


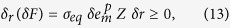


where the variable 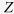 is 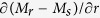. Now, we are ready to introduce the variable 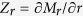, which quantifies the rate of plastic work that is stored in the evolving ensemble of slip systems. The available experimental data suggests that plastic deformation slows down the storage^11^. The condition (13) suggests that a homogeneous deformation produces stable microstructural reconfigurations. The homogeneity can be violated when localized shear bands are formed 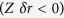, or the material experiences other damages. The formation of localized shear bands promotes realignment of slip systems. In such a case, the parameter 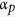 should take negative values. This condition can be constructed by assuming that the parameter 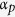 is a function of plastic strain and it takes negative values at a critical state of plastic deformation. Also, a strong dynamic excitation may trigger unstable microstructural evolution. We paraphrase the argument by Prigogine^21^ and state that the instability results from the interplay of three aspects which are always linked in a dissipative material: there is the mechanism of inelastic flow, there are the microstructural reconfigurations, and there are the flow fluctuations, which trigger the instability. The role of such fluctuations in a plastically deformed material has been discussed in ref. 22.

### Power-law viscoplasticity

In our approach, the rate of the microscopic plastic strain is expressed in terms of the current stress. We propose a power-law constitutive relation, which couples the rate of the micro-plastic equivalent strain with the equivalent stress


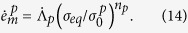


Strength 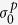 is a temperature sensitive quantity and, therefore, the actual strength is 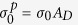, while the athermal strength 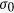 is a material constant. We correct the power-law rate relation in (14) and introduce a rate-sensitive factor 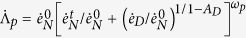, where 
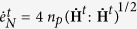
 , the parameter 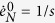 is the reference rate, and 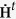 is the rate of total strain. The term 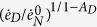 describes the contribution of diffusional flow, where the parameter 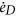 is a constant and is in the order of 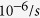. The function *A*_*D*_ has already been derived (Eqn. 1) and the exponent *ω_p_* is a constant slightly smaller from one. At high temperatures and/or very low strain rates, the term 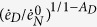 affects the flow process defined in (14). At the initial stage of plastic deformation, the rate of elastic strain dominates 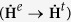 and, therefore, we have 
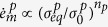
. When the rate of plastic strain controls the deformation process 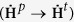, the strain rate sensitivity is 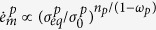. Consequently, the material experiences a smooth strain rate transition from 
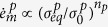
 to a nearly strain rate insensitive plastic flow 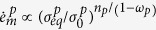. In this construction, the model captures diffusional flow, power-law creep and plasticity. It is worth mentioning that similar power-law expressions have already been used for metals^23^,^24^ and frictional materials^25^. Elastic material properties complete the constitutive description. We use the rule of the strain rate additivity 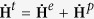 and, then, the rate of stress is 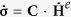, where the fourth order elastic tensor is **C**.

## Discussion

### Model validation

**Figure 2:**
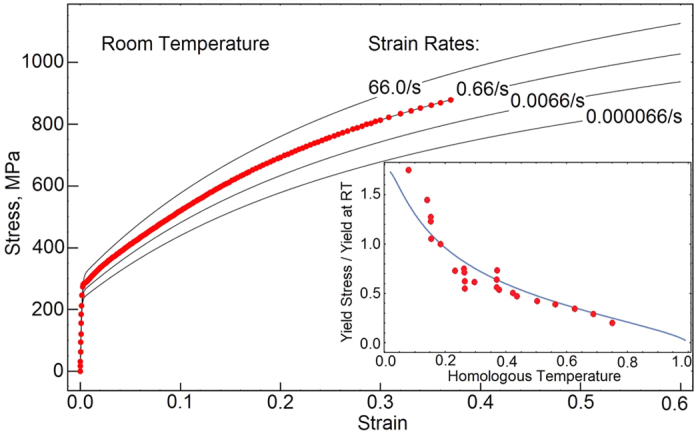
Uniaxial stress versus strain in tension is plotted for four different strain rates and at room temperature. Red dots represent experimental data. The embedded plot shows yield stress constructed in terms of temperature. The prediction is compared with experimental data gathered from refs 26, 27, 28.

**Figure 3:**
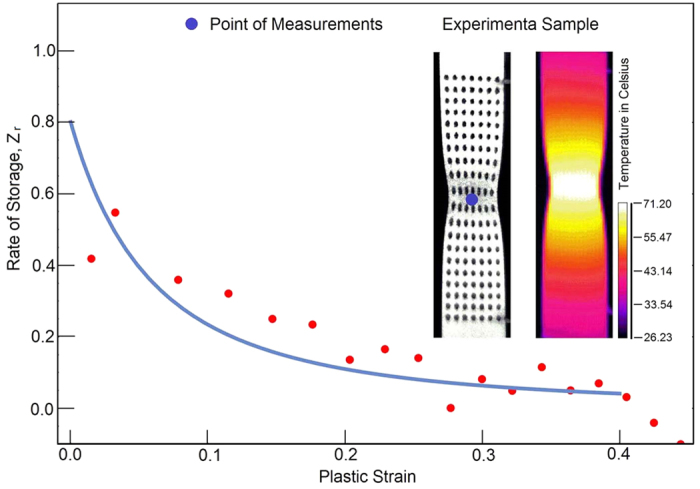
The rate of energy storage is plotted as a function of plastic strain. Red dots represent our experimental data obtained from the simultaneous measurements of infrared and visible cameras.

**Figure 4:**
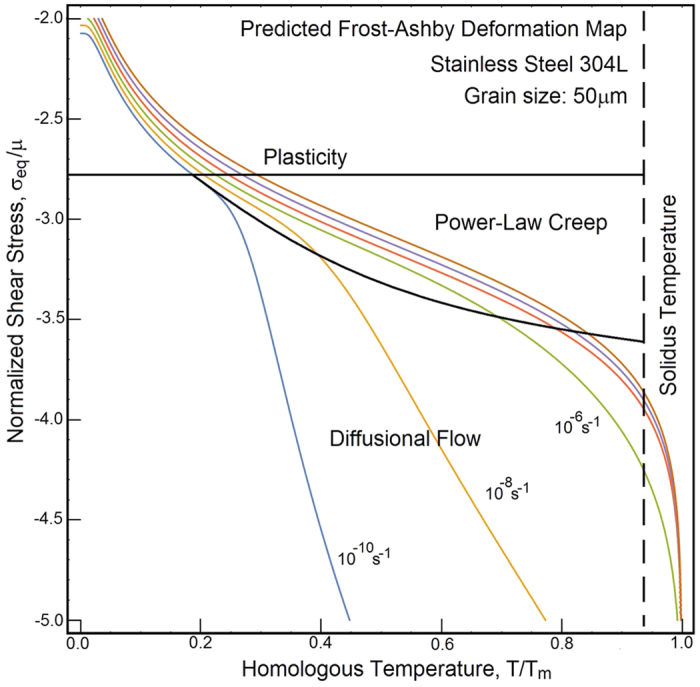
The mechanisms-based model is used for the prediction of the Frost-Ashby deformation map for stainless steel 304L (ref. 32, p. 66). The chemical composition of the steel is presented in Table 2. Solid lines represent the material responses at constant strain rate plotted as a function of the normalized shear stress and homologous temperature.

We have four groups of independently verifiable parameters. First group constitutes elastic properties 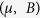. The second describes the thermally activated processes 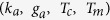. The average Schmid factor is defined in terms of two parameters 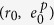 and, then, the rate of slip reconfigurations is controlled by the parameter 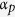. Lastly, the power-law flow rules are determined in terms of four parameters 
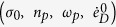
. The thermally activated processes are calibrated against experimental data, where yield stress is plotted as a function of temperature. The average Schmid factor controls the rate of plastic hardening and the rate of the energy storage. The flow rules determine the stress-strain rate sensitivity. We calibrate the model for stainless steel 304L and the parameters are listed in the Table 1. The model is coded and figures are constructed using the Wolfram software Mathematica. First, we compare the predicted and experimentally obtained stress-strain responses at strain rate 0.66/*s* and room temperature, Fig. 2. Red dots in the plot represent the experimental data. We also show the material’s sensitivity to strain rates ranging from 6.610^−5^/*s* to 66.0/*s*. On the same plot, we include the normalized yield stress plotted as a function of temperature. Yield stress is normalized with respect to yield at room temperature. Again, the dots represent published experimental data^26^,^27^,^28^. Both the predictions in Fig. 2 are matching the experimental data, though it is understandable that the data gathered from multiple sources exhibits some scatter. Next, we compare the experimentally measured rate of the energy storage and compare it with our predictions 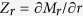, Fig. 3. The scatter of measurements results from the heat fluctuations, which occur both in time and space. Such fluctuations are caused by stochasticity of the dislocation multiplication, motion and storage. Similar responses are obtained for 2024-T3 aluminum alloy and α-titanium; both of the materials are subjected to quasi-static and dynamic loading^29^. In all the cases^29^,^30^,^31^, the rate of storage decreases as the material is plastically deformed. Our model correctly predicts the slowdown of the storage. As we indicated earlier, the formation of shear bands should reverse the trends. The power-law viscoplasticity captures the behaviors from diffusional flow, power-law creep up to the plastic flow. We demonstrate these deformation mechanisms by constructing the Frost-Ashby deformation map^32^, Fig. 4. Our map captures the main features of the behavior within a broad range of strain rates and temperatures.

Our motivation has been to capture the relevant micro-mechanisms of plastic flow, while retaining the simplicity of the phenomenological material description. In this manner, we bridge the phenomenology with the relevant mechanisms of plastic deformation. We do not identify independent crystallographic orientations and, instead, apply weight to each 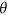 orientation. Thus, in our approach slip occurs along a broad spectrum of planes and, then, the plastic flow is projected onto the plane of maximum shear stress. The slip reconfigurations are detectable in our measurements of the energy storage rate. We have shown that these reconfigurations are stable in a homogeneously deformed material. However, the processes may become unstable in the presence of localized shear bands. The latter are triggered by strong spatial and temporal stress fluctuations discussed in ref. 22. We complete the approach by introducing the description for the thermally activated motion of dislocations, where two characteristic temperatures are introduced. The transition temperature *T*_*c*_ differentiates the behavior between the power-law creep mechanism and the low-rate sensitive dislocation glide. The melting temperature *T*_*m*_ is introduced for a proper description of diffusional flow. Also, we propose a generalized model for the power-law flow, where we capture the inelastic behaviors associated with diffusional flow, power-law creep and plasticity. We validate the approach by developing a constitutive model for stainless steel 304L. The material’s chemical composition is designed for impeding the deformation-induced martensitic transformation.

## Methods

### Experimental procedure

As mentioned above, a part of the plastic work is dissipated as heat and the rest of it increases internal energy. In the area of the strain localization, the distribution of the strain rates is not uniform and, therefore, a non-uniform temperature field is observed on the surface of the specimen^31^. Evolution of the temperature field during the deformation process is recorded by a high sensitivity IR thermography system. The average temperature of the selected section of the specimen is plotted as a function of time. Note that the section is positioned in such a manner to avoid the influence of the specimen edges on the measured temperature values. The initial volume of the section is equal to (1.3 × 1.3 × 2) mm^3^ and the initial cross-section is (1.3 × 2) mm^2^. The size of the section is changing during the tensile deformation. These changes are monitored by tracking the section’s center in the visible range by means of the CCD camera. All these data have been used to calculate heat generated during short time interval in the section. The interval is the reciprocal of the measurement frequency. The experiments are performed on austenitic stainless steel with chemical composition shown in Table 2. This composition is developed to mitigate the phase transformation during the deformation process. The strips with the cross-section 25 mm × 4 mm are initially annealed at 1,050 °C, water quenched and 50% cold rolled. Then, the specimens for tensile testing are cut to the right shape using an electro-erosion machining. Prior to testing, the specimens are annealed for 1 h at 1,100 °C and water quenched to produce a homogeneous microstructure with a mean grain size about 50 μm and are electro-polished. We use MTS 858 testing machine and apply tensile deformation with the displacement rate, which corresponds the strain rate equal to 0.66/s. In the course of the deformation process, the infrared and visible range image sequences are recorded simultaneously using the infrared Phoenix and pco. 1200 hs cameras. The measurement frequency of both cameras is equal to 538 Hz. This frequency is sufficient to avoid the contribution of heat transfer between neighboring sections. The analysis of the displacement field (based on markers tracking) and the appropriate temperature distributions is performed using MATLAB software.

## Additional Information

**How to cite this article**: Zubelewicz, A. and Oliferuk, W. Mechanisms-based viscoplasticity: Theoretical approach and experimental validation for steel 304L. *Sci. Rep.* **6**, 23681; doi: 10.1038/srep23681 (2016).

## Figures and Tables

**Table 1:** Material constants for steel 304L.

Elasticity		Thermal Activation				Average Schmid Factor			Power-Law Viscoplasticity			
*μ*	*B*	*k*_*a*_	*g*_*a*_	*T*_*c*_	*T*_*m*_	*r*_*0*_	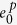	*α*_*p*_	*σ*_0_	*n*_*p*_	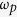	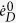
*MPa*	*MPa*	–	–	*K*	*K*	–	*mm*/*mm*	–	*MPa*	–	–	1/*s*
8110^3^	12510^3^	2/3	2/3	530	1600	0.1	0.013	2.56/*π*	550	1	0.98	510^−6^

**Table 2:** Chemical composition (wt. %).

C	Mn	Si	P	S	Cr	Ni	W	Mo	Cu	V	Ti	Fe
0.05	1.35	1.0	0.016	0.008	18.58	17.3	0.025	0.02	0.04	0.03	0.013	Balance
